# Low amounts of dietary fibre increase *in vitro* production of short-chain fatty acids without changing human colonic microbiota structure

**DOI:** 10.1038/s41598-017-18877-8

**Published:** 2018-01-11

**Authors:** Daisuke Sasaki, Kengo Sasaki, Naoko Ikuta, Takahiro Yasuda, Itsuko Fukuda, Akihiko Kondo, Ro Osawa

**Affiliations:** 1 Graduate School of Science, Technology and Innovation, Kobe University, 1-1 Rokkodai-cho, Nada-ku, Kobe, Hyogo 657-8501 Japan; 2 Graduate School of Medicine, Kobe University, 7-5-2 Kusunoki-cho Chuo-ku, Kobe, Hyogo 650-0017 Japan; 3 Clinical & Translational Research Center, Kobe University Hospital, 7-5-2 Kusunoki-cho Chuo-ku, Kobe, Hyogo 650-0017 Japan; 4 Department of Bioresource Science, Graduate School of Agricultural Science, Kobe University, 1-1 Rokkodai-cho, Nada-ku, Kobe, Hyogo 657-8501 Japan; 5 Research Center for Food Safety and Security, Graduate School of Agricultural Science, Kobe University, 1-1 Rokkodai-cho, Nada-ku, Kobe, Hyogo 657-8501 Japan; 6 RIKEN Center for Sustainable Resource Science, 1-7-22 Suehiro-cho, Tsurumi-ku, Yokohama, Kanagawa 230-0045 Japan

## Abstract

This study investigated the effect of various prebiotics (indigestible dextrin, α-cyclodextrin, and dextran) on human colonic microbiota at a dosage corresponding to a daily intake of 6 g of prebiotics per person (0.2% of dietary intake). We used an *in vitro* human colonic microbiota model based on batch fermentation starting from a faecal inoculum. Bacterial 16S rRNA gene sequence analysis showed that addition of 0.2% prebiotics did not change the diversity and composition of colonic microbiota. This finding coincided with results from a clinical study showing that the microbiota composition of human faecal samples remained unchanged following administration of 6 g of prebiotics over seven days. However, compared to absence of prebiotics, their addition reduced the pH and increased the generation of acetate and propionate in the *in vitro* system. Thus, even at such relatively low amounts, prebiotics appear capable of activating the metabolism of colonic microbiota.

## Introduction

Prebiotics are defined as “a selectively fermented ingredient that results in specific changes in the composition and/or activity of the gastrointestinal microbiota, thus conferring benefit(s) upon host health”^1^. Dietary prebiotics are not digested in the human small intestine and enter the colon, where they are fermented by the gut microflora^2,3^.

Indigestible dextrins are composed of a glucose polymer and are considered as prebiotic fibre functional ingredients^2^. Short-chain fatty acids (SCFAs) are fermentation products of indigestible dextrin that exert important health functions, such as regulating the absorption of water and minerals and reducing colonic pH to inhibit potential pathogens and promote growth of beneficial bacteria^3^. Recently, an *in vitro* study demonstrated that at a concentration of 1.25% (wt/vol), dextrins from maize or wheat stimulate the growth of beneficial microorganisms such as those from the genera *Bifidobacterium* and *Lactobacillus*, and the phylum Bacteroidetes^4–6^. Cyclodextrins (CDs) are industrially produced enzyme-modified starch derivatives that can be used also as food additives^7^. CDs are cyclic oligosaccharides consisting of six (αCD), seven (βCD), or eight (γCD) α-1,4-linked glycopyranose units, with a hydrophilic hydroxyl group on their outer surface and a hydrophobic cavity in their centre. In these CDs, αCD is widely used as a water-soluble dietary fibre^8^. Addition of 1.5% αCD to mice has been shown to decrease the numbers of caecal bacteria belonging to the genus *Clostridium* ^9^. In contrast, dextran is produced by lactic acid bacteria of the genera *Leuconostoc*, *Streptococcus*, *Lactococcus*, and *Lactobacillus* ^10^. *In vitro* experiments have shown that administration of 1-kDa dextrans (linear and α-1,2 branched) at a concentration of 1% could selectively increase the number of bacterial cells belonging to the genus *Bifidobacterium* ^11,12^. However, given that the daily Japanese human diet is approximately 3 L (3,000 g)^13,14^, the amounts of prebiotics administered in previous studies (same or more than 1%, i.e., ≥30 g/day) were too high and should be decreased to reflect a more realistic dosage.

*In vitro* models do not pose the same ethical constraints as *in vivo* human trials and allow dynamic sampling to study microbial activity *in vivo*. Thus, *in vitro* batch fermentation systems inoculated with human faecal matter to mimic the human digestive tract environment have been applied to measure production of SCFAs^15^ and test the modulatory effect of potential prebiotics on human gut microbiota^16–18^. By carefully constructing the necessary anaerobic conditions, we previously developed an *in vitro* batch fermentation system (hereafter referred to as Kobe University Human Intestinal Microbiota Model, KUHIMM) that is capable of hosting more than 500 microbial species found in a human faecal inoculum and can effectively mimic human colonic microbiota^13^. The number of microbial species in this system was similar to the that (400–1000) previously reported in human faecal samples^19,20^. Our KUHIMM was able to reproduce the bifidogenic effects of prebiotic materials (i.e., fructo-, galacto-, iso-malto- and xylo-oligosaccharides), in line with the results from human clinical trials^21^.

The aim of this study was to assess the effect of relatively low amounts of three different prebiotics, i.e., indigestible dextrin (DEX), α-cyclodextrin (αCD), and dextran (DXR), on human colonic microbiota using our KUHIMM system (Table 1). The prebiotic concentration was set to 0.2%, corresponding to 6 g prebiotics per 3 L of daily dietary intake^13,14^, and was sufficiently low to avoid the occurrence of any intestinal discomfort. The bacterial composition in the KUHIMM following prebiotic treatment was evaluated by next-generation sequencing (NGS) and compared with that in the absence of prebiotics. Similarly, we compared also pH changes and production of SCFAs. In addition, a small human trial was performed with a daily intake of 6 g of DEX or αCD to compare the microbiota composition in actual human faeces with that in the KUHIMM.

**Table 1:** Prebiotics used in this study.

Prebiotics	Glucosidic linkages	Molecular weight
Indigestible dextrin (DEX)	α-1,4*, α-1,6, α-1,2, α-1,3	1,800–2,000
α-Cyclodextrin (αCD)^#^	α-1,4*	972
Dextran (DXR)	α-1,6*, α-1,4	32,000–45,000

*Predominant linkage.

^#^α-cyclodextrin is a cyclic oligosaccharide consisting of six glucopyranose units.

## Results

### Prebiotics reduce the pH in the KUHIMM

**Figure 1:**
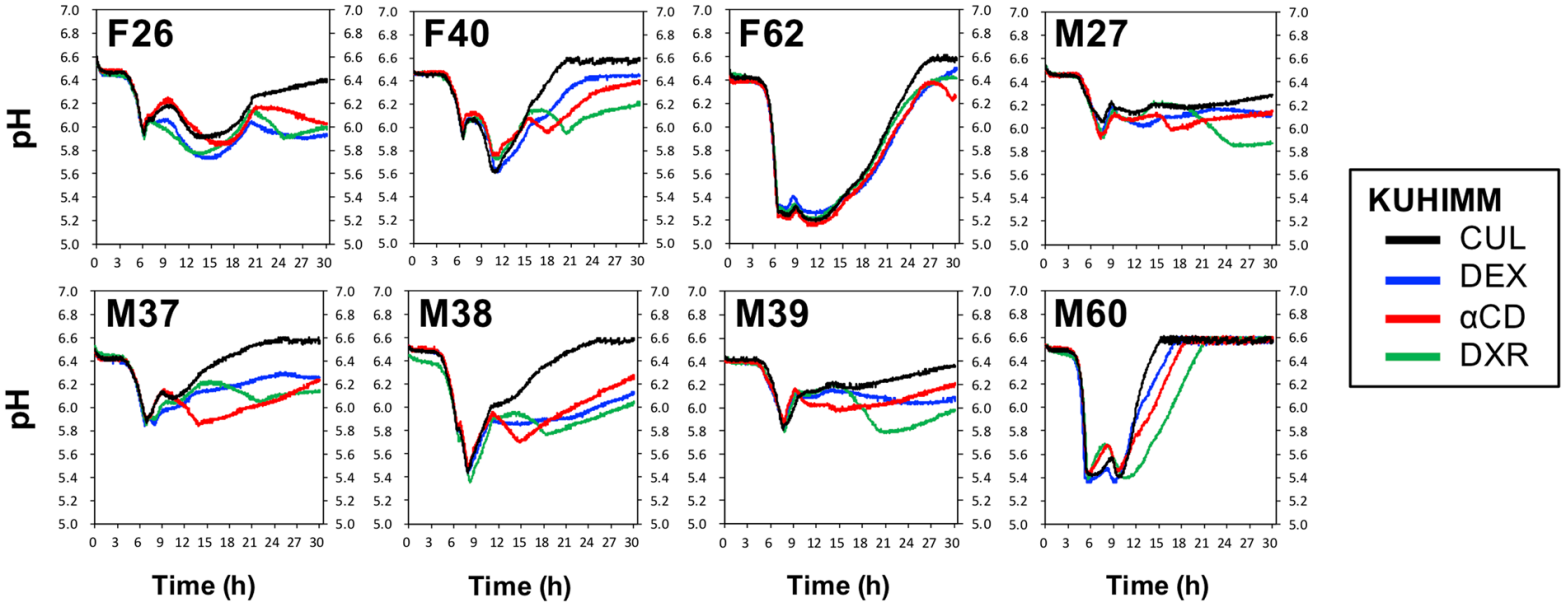
pH profiles obtained during fermentation in the KUHIMM with and without 0.2% prebiotics. Fermentation was initiated by inoculating each of the human faecal samples (designated as F26, F40, F62, M27, M37, M38, M39, and M60). pH profiles without prebiotics (CUL) and with 0.2% indigestible dextrin (DEX), α-cyclodextrin (αCD), or dextran (DXR) are shown with black, blue, red, and green lines, respectively.

The KUHIMM was operated by adding 0.2% DEX, αCD, or DXR, and each of eight human faecal samples (designated as F26, F40, F62, M27, M37, M38, M39, and M60) was used as the inoculum. A control that included no prebiotics was also prepared. During the fermentation, pH transition occurred due to production of mainly SCFAs and ammonia (Supplementary Fig. S1). Continuous monitoring (Fig. 1) revealed an overall reduction in pH in the later phase of fermentation in the presence of prebiotics compared to the control. The lower pH reflected changes in environmental conditions resulting from the interplay between prebiotics and human gut microbiota.

### Addition of 0.2% prebiotics does not alter microbial diversity

DNA was extracted from human faeces and from KUHIMM samples collected at 30 h of fermentation. In all KUHIMM samples, eubacterial copy numbers, evaluated by quantitative PCR, reached 9.15 × 10^10^ copies/mL (Supplementary Table S1) and were comparable to the reported cell densities in human faeces (approximately 10^11^/wet-g)^22^.

NGS was used for bacterial 16S rRNA gene sequence analysis of faecal samples and corresponding cultures with and without prebiotics. In total, 5,922,873 quality reads with an average of 148,072 reads were obtained (Table 2). The numbers of operational taxonomic units (OTUs) and α-diversity values (Chao1, Shannon index, and Simpson index) were not lower in cultures treated with or without prebiotics than in faecal samples (Kruskal-Wallis test). Thus, as described previously, bacterial numbers in human faeces were maintained in the corresponding cultures of our KUHIMM^13^. There were fewer OTUs in cultures treated with DEX and αCD than in control cultures (*P* < 0.05, Kruskal-Wallis test); however, OTU numbers were higher in the presence of DRX than in the absence of prebiotics (*P* < 0.05, Kruskal-Wallis test). Chao1 values, which indicate species richness, were lower in cultures with DEX and αCD than in controls (*P* < 0.05, Kruskal-Wallis test), but were not significantly different between DRX-treated cultures and controls (*P* > 0.05, Kruskal-Wallis test). Moreover, the Shannon and Simpson indexes were not significantly different between cultures containing prebiotics and controls (*P* > 0.05, Kruskal-Wallis test). Therefore, species diversity in our KUHIMM did not change following addition of 0.2% prebiotics.

**Table 2:** Summary of 16S rRNA gene sequencing data and α-diversity values (Chao1 estimator, Shannon index, and Simpson index).

	KUHIMM (n = 8)				
	FEC	CUL	DEX	αCD	DXR
Read counts	123,622 ± 29,146	166,869 ± 35,152	151,911 ± 30,120	79,656 ± 12,693	218,300 ± 42,461
Observed OTUs	1,168 ± 291	1,525 ± 204*	1,294 ± 167^#^	1,083 ± 151^##^	1,954 ± 326^#^
Chao1	1,834 ± 441	2,362 ± 321*	2,024 ± 253^#^	1,896 ± 255^#^	2,751 ± 510
Shannon index	5.39 ± 0.26	5.24 ± 0.25	5.14 ± 0.32	5.10 ± 0.27	5.31 ± 0.36
Simpson index	0.94 ± 0.01	0.94 ± 0.02	0.94 ± 0.02	0.93 ± 0.01	0.94 ± 0.02

Eight human faeces samples (FEC), corresponding cultures (CUL), and corresponding cultures treated with 0.2% indigestible dextrin (DEX), α-cyclodextrin (αCD), or dextran (DXR) were sampled at 30 h of fermentation.

The values show the mean ± standard deviation.

Statistical differences between samples were evaluated with respect to observed operational taxonomic units (OTUs), Chao1 estimator, Shannon index, and Simpson index.

Asterisks (*) indicate significant differences between faeces from eight healthy subjects and corresponding cultures without or with prebiotics (*P < 0.05 and **P < 0.01, Kruskal-Wallis test).

Pounds (^#^) indicate significant differences between cultures with and without prebiotics (^#^ *P* < 0.05 and ^##^ *P* < 0.01, Kruskal-Wallis test).

### Addition of 0.2% prebiotics does not change microbial composition

**Figure 2:**
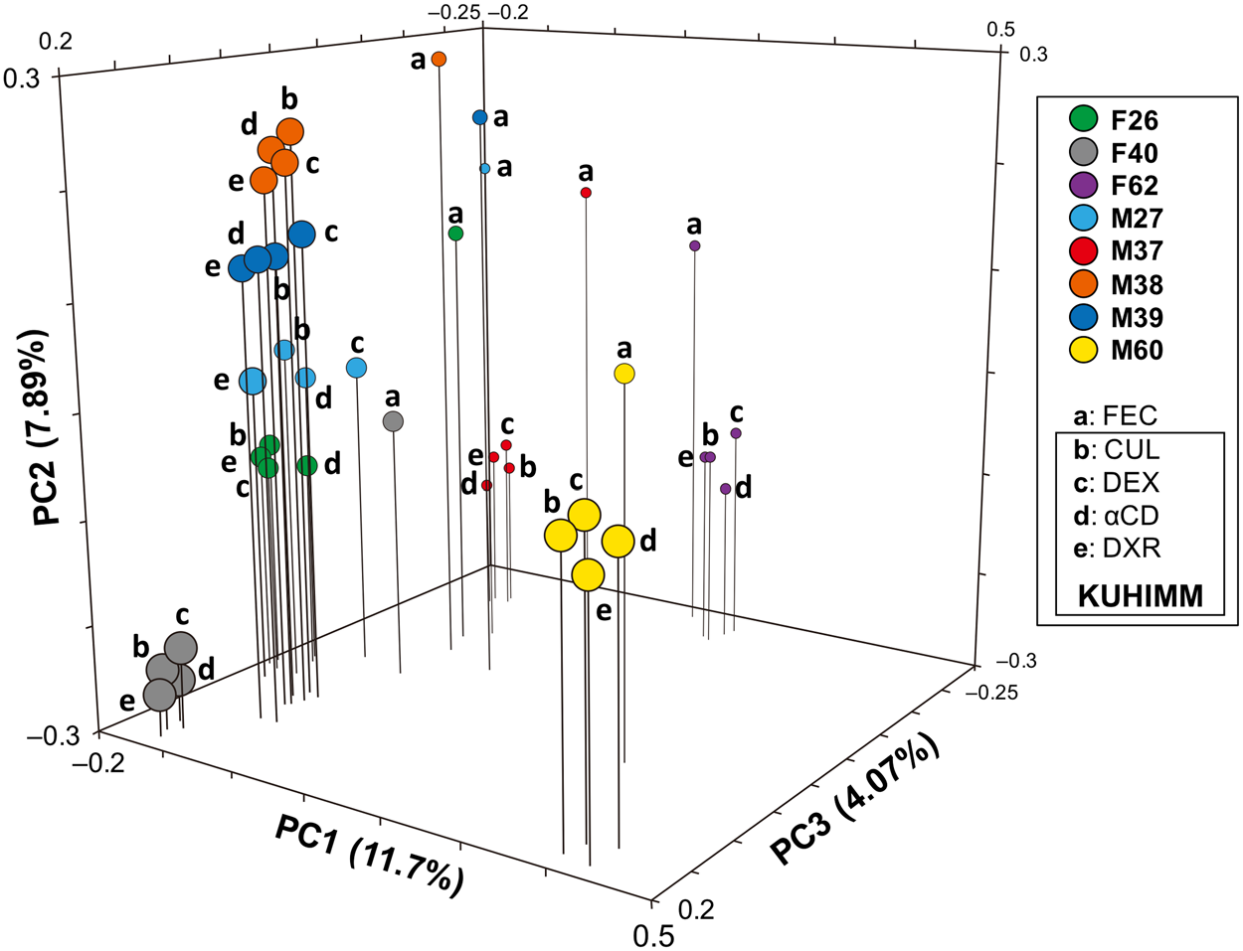
Principal coordinate analysis (PCoA) of 16S metagenomics data of bacterial species in eight human volunteers’ faeces (designated as F26, F40, F62, M27, M37, M38, M39, and M60) and corresponding cultures with and without 0.2% prebiotics: indigestible dextrin (DEX), α-cyclodextrin (αCD), and dextran (DXR). Cultures were sampled at 30 h after initiation of fermentation. (**a**) Human faeces (FEC); (**b**) corresponding culture without prebiotics (CUL); (**c**) corresponding culture with 0.2% DEX; (**d**) corresponding culture with αCD; (**e**) corresponding culture with DXR.

Principal coordinate analysis (PCoA) of unweighted UniFrac distances revealed that microbiota in faecal samples shifted in the same direction as in the corresponding cultures without prebiotics (*P* = 0.454, multivariate analysis of variance (MANOVA)) (Fig. 2). No significant changes were detected between the microbiota of cultures with and without prebiotics (DEX: *P* = 0.571; αCD: *P* = 0.986; DXR: *P* = 0.996, MANOVA).

**Figure 3:**
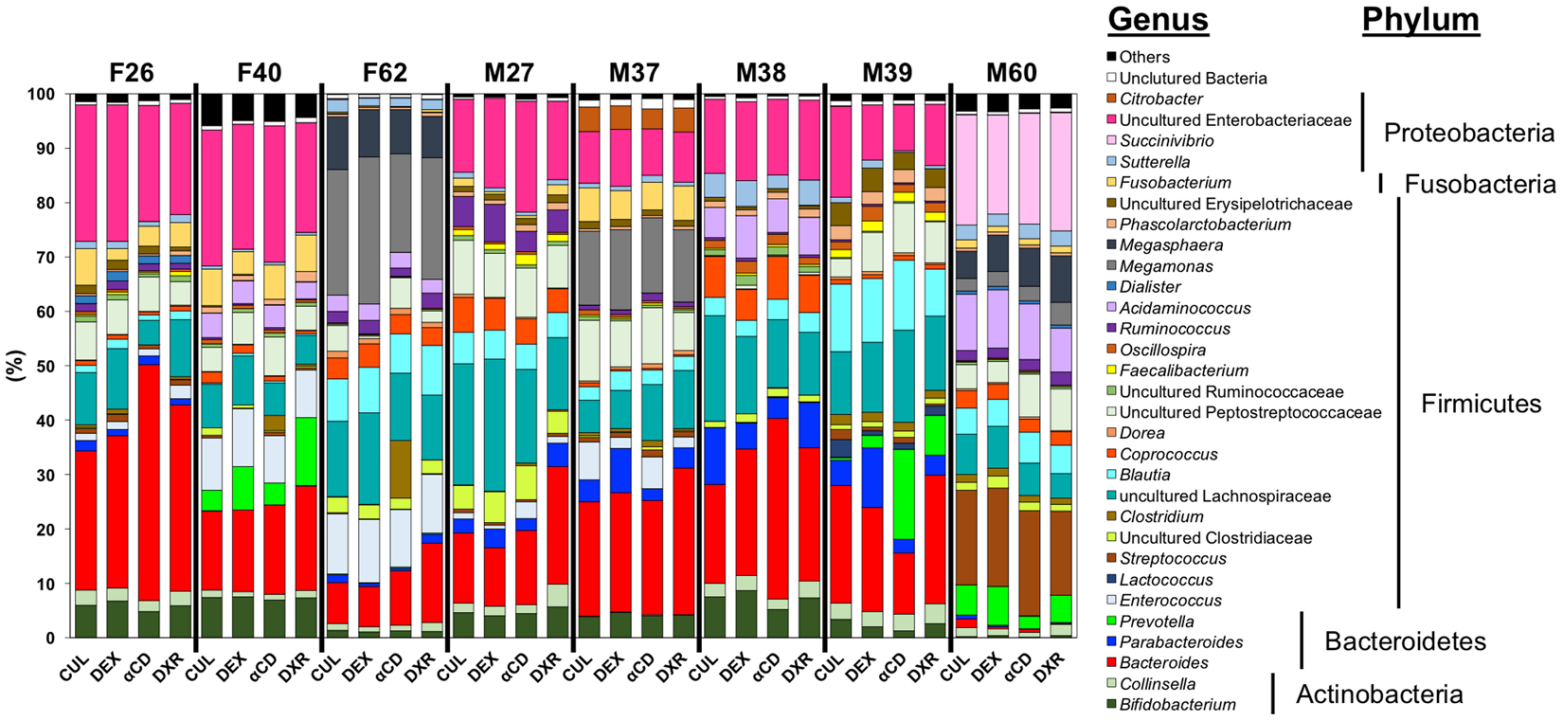
Genus-level compositional view of bacteria in cultures after 30 h of fermentation. One each of eight human faecal samples (designated as F26, F40, F62, M27, M37, M38, M39, and M60) was used as the inoculum in each KUHIMM. Samples were obtained from fermentation cultures without prebiotics (CUL) and cultures with 0.2% indigestible dextrin (DEX), α-cyclodextrin (αCD), or dextran (DRX) at 30 h after initiation of fermentation. Genera of lower abundance (<1.0%) and lower similarity (<97%) were included in Others and Uncultured Bacteria, respectively.

Bacterial composition was compared in cultures with and without prebiotics (Fig. 3). Approximately 99% of the total bacterial abundance was assigned to five phyla Actinobacteria, Bacteroidetes, Firmicutes, Fusobacteria, and Proteobacteria. Relative abundances of the genera *Bifidobacterium*, *Collinsella*, *Bacteroides*, *Parabacteroides*, *Prevotella*, *Enterococcus*, *Lactococcus*, *Streptococcus*, uncultured Clostridiaceae, *Clostridium*, uncultured Lachnospiraceae, *Blautia*, *Coprococcus*, *Dorea*, uncultured Peptostreptococcaceae, uncultured Ruminococcaceae, *Faecalibacterium*, *Oscillospira*, *Ruminococcus*, *Acidaminococcus*, *Dialister*, *Megamonas*, *Megasphaera*, *Phascolarctobacterium*, uncultured Erysipelotrichaceae, *Fusobacterium*, *Sutterella*, *Succinivibrio*, uncultured Enterobacteriaceae, and *Citrobacter* did not differ significantly between cultures with and without prebiotics (*P* < 0.05, Kruskal-Wallis test). Thus, bacterial compositions in our KUHIMM did not change following addition of 0.2% prebiotics.

### Addition of 0.2% prebiotics increases production of SCFAs

**Figure 4:**
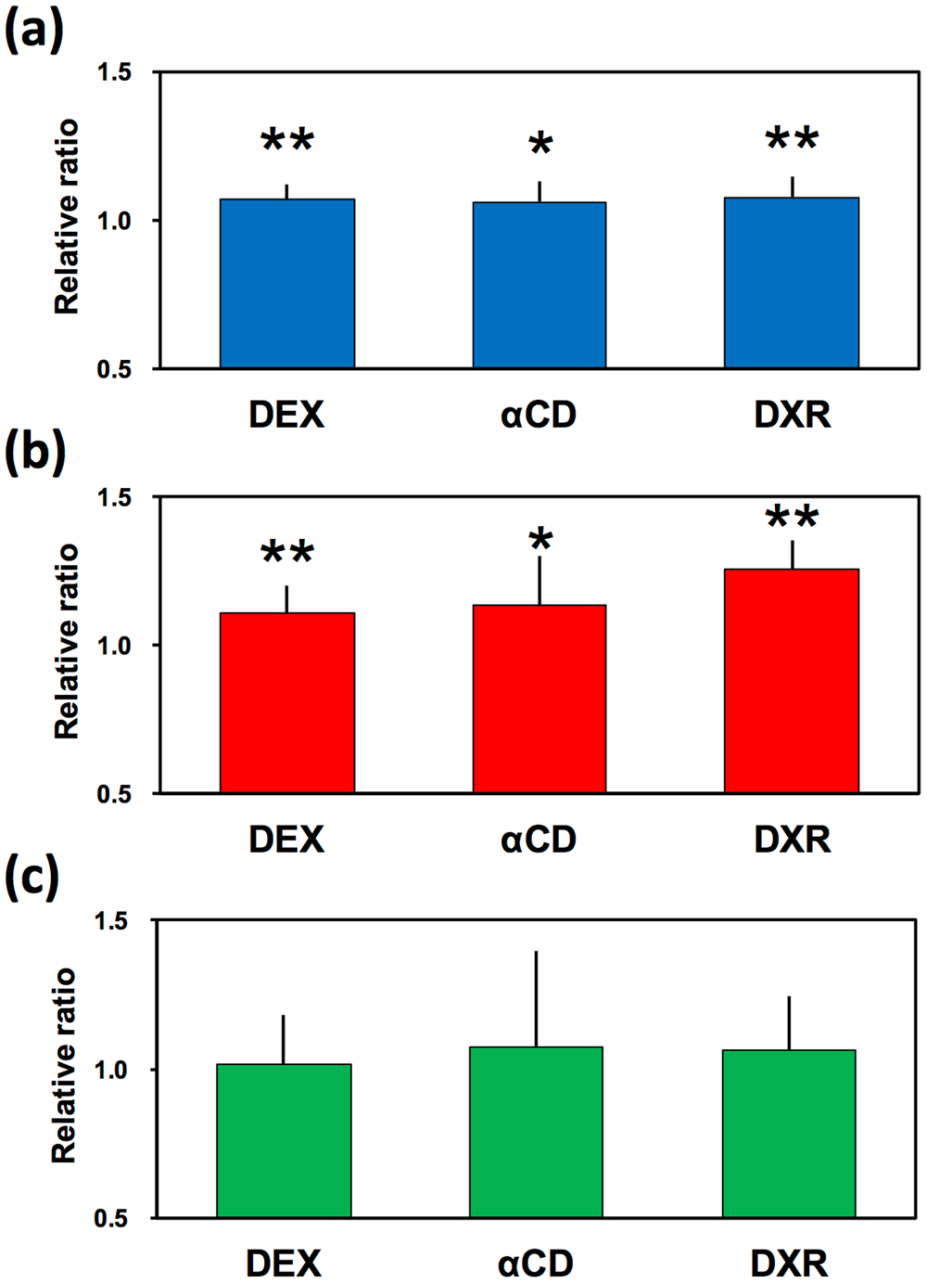
Changes in production of (**a**) acetate, (**b**) propionate, and (**c**) butyrate at 30 h after initiation of fermentation in the KUHIMM supplemented with 0.2% prebiotics: indigestible dextrin (DEX), α-cyclodextrin (αCD), and dextran (DXR). Changes are presented as the ratio of the concentrations in the KUHIMM with prebiotics normalised to those without prebiotics. Asterisks (*) indicate significantly different values (*P < 0.05 and **P < 0.01) (n = 8) using Dunnett test. Error bars show the standard deviation of the mean.

SCFAs are the end-products of microbial fermentation in the colon and act as signalling molecules between gut microbiota and the host, with important implications for host health^3,23^. The effect of adding 0.2% prebiotics on SCFA production in the KUHIMM was evaluated (Fig. 4). At 30 h after initiating fermentation and in the absence of prebiotics, the production of acetate, propionate, and butyrate was 101.0 ± 23.2 mM, 35.9 ± 9.69 mM, and 26.9 ± 11.5 mM, respectively. The concentration of lactate was lower than 0.02 mM. Interestingly, the concentrations of acetate and propionate were significantly higher following addition of prebiotics (*P* < 0.05, Dunnett test), whereas that of butyrate remained unchanged.

### The effect on faecal microbiota composition is confirmed by a human intervention study

**Figure 5:**
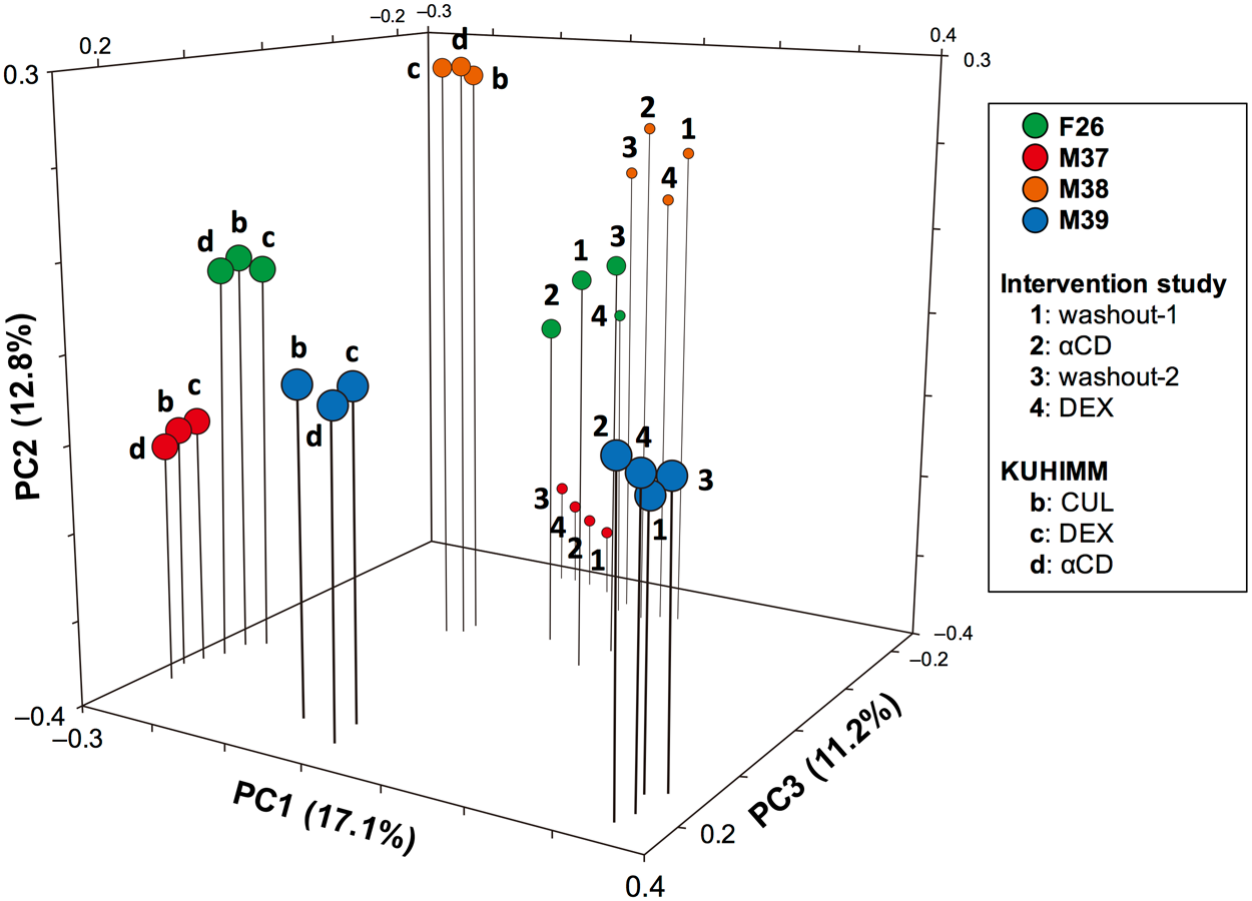
Principal coordinate analysis (PCoA) of 16S metagenomic data of bacterial species in human faecal samples obtained from a human intervention study. Four human volunteers (designated as F26, M37, M38, and M39) participated in this study. In one subject, two faecal samples (designated as washout-1 (plot 1) and washout-2 (plot 3)) were collected after the subjects drank water for 14 days and another two faecal samples (designated as αCD (plot 2) and DEX (plot 4)) were collected after the subjects consumed α-cyclodextrin or indigestible dextrin, respectively, for seven days. In addition, PCoA plots, which were obtained from the KUHIMM without prebiotics (CUL (plot b)) and with indigestible dextrin (DEX (plot c)) or α-cyclodextrin (αCD (plot d)) (already shown in Fig. 2), are also presented. Faecal samples from the subjects participating in the human intervention study were used to inoculate the cultures in the KUHIMM.

To compare the effect of a daily intake of dietary fibre on healthy human microbiota with data from the KUHIMM, we performed a preliminary, small scale, open-label clinical study. To this end, we investigated each faecal sample obtained from four human volunteers (F26, M37, M38, and M39), who ate a daily amount of 6 g of DEX or αCD for seven days. Before eating prebiotics, the composition of human gut microbiota was calibrated by drinking 200 mL of water for 14 days (washout-1) (Supplementary Fig. S2). Faecal samples were collected four times during the final days of eating prebiotics and drinking water. NGS analysis showed that species (OTU) numbers and microbial diversity (α-diversity) did not differ significantly between faeces collected after eating prebiotics or after drinking water (*P* > 0.05, Kruskal-Wallis test) (Table 3). Similar to the results in the KUHIMM, PCoA analysis revealed that microbiota compositions clustered equally in the four faecal samples (two with prebiotics and two without) from the same individual (Fig. 5). Thus, eating 6 g of DEX or αCD for seven days did not significantly affect the microbiota composition in the human colon. The results obtained in the human intervention study coincided with the findings in the KUHIMM.

**Table 3:** Summary of 16S rRNA gene sequencing data and α-diversity values (Chao1, Shannon index, and Simpson index) in faecal samples obtained from a human intervention study.

	Human intervention study (n = 4)			
	washout-1	αCD	washout-2	DEX
Read counts	116,239 ± 38,393	129,196 ± 42,459	138,811 ± 48,413	125,079 ± 42,432
Observed OTUs	1,518 ± 288	1,548 ± 471	1,534 ± 460	1,620 ± 527
Chao1	2,416 ± 558	2,459 ± 675	2,457 ± 538	2,530 ± 752
Shannon index	5.70 ± 0.36	5.35 ± 0.69	5.53 ± 0.70	5.64 ± 0.67
Simpson index	0.95 ± 0.01	0.92 ± 0.04	0.94 ± 0.03	0.95 ± 0.02

Four human volunteers participated in this study. Faecal samples were obtained after the subjects drank water for 14 days (washout-1 or washout-2) and ate α-cyclodextrin (αCD) or indigestible dextrin (DEX) for seven days.

The values show the mean ± standard deviation.

No statistical difference was detected between faecal samples (washout-1, αCD, washout-2, and DEX) in terms of observed operational taxonomic units (OTUs), Chao1, Shannon index, and Simpson index (Kruskal-Wallis test).

## Discussion

The aim of the present study was to investigate the impact of a low dosage (0.2%) of prebiotics (DEX, αCD, or DXR) on human colonic microbial ecology and metabolic end-products using an *in vitro* batch fermentation system, KUHIMM. We mimicked a daily intake of 6 g of prebiotics, assuming that the total Japanese human daily intake of foods and beverages is 3000 g^14^. Our *in vitro* and *in vivo* experiments suggest that a low dosage of prebiotics did not significantly affect the colonic microbiota composition. This contrasts with previous reports where a higher intake of prebiotics such as wheat dextrin (10–20 g/day in humans)^24,25^, αCD (1.5% in mice)^9^, and DXR (1–10% in an *in vitro* system)^26,27^ substantially changed the colonic microbiota composition. However, addition of prebiotics reduced the pH and promoted the generation of acetate and propionate even at low concentrations, as revealed by the *in vitro* experiments.

This increase in acetate and propionate is in line with previous findings on the administration of wheat dextrin^28^, αCD^29^, and DXR^26^. Bacteria belonging to the phylum Bacteroidetes, which make up a large proportion of the intestinal microbiota, are known to produce mainly propionate along with acetate^30–32^. The phylum Bacteroidetes harbours a very broad saccharolytic potential^33^ and plays an important role in fermenting indigestible carbohydrates in the proximal colon^30^. Here, the relative abundance of this phylum correlated positively with the prevalence of propionate among total SCFAs (Supplementary Fig. S3), as observed previously in human faeces^34,35^. In addition, bacteria belonging to the genus *Bifidobacterium*, which were abundant in the phylum Actinobacteria in our system, are known to produce acetate^36,37^. Not surprisingly, the relative abundance of Actinobacteria showed a positive correlation with the proportion of acetate among total SCFAs (Supplementary Fig. S3). However, the cell abundance of phyla Bacteroidetes and Actinobacteria did not show a significant difference between control cultures and cultures with prebiotics, considering that the total number of eubacteria and relative abundance of these phyla were similar in the cultures, although cell abundance reportedly correlates with metabolic activity, i.e., rates of fermentation^34^. Therefore, it was expected that prebiotics such as DEX, αCD, and DXR could potentially increase the metabolic activity of bacteria belonging to the phyla Bacteroidetes and Actinobacteria, irrespective of the lack of increase in growth at low dosage amounts. In contrast, bacteria belonging to the phylum Firmicutes are known to produce butyrate as the primary metabolic end-product^30^. Thus, the relative abundance of the phylum Firmicutes correlated positively with the proportion of butyrate among total SCFAs (Supplementary Fig. S3). Low amounts of the tested prebiotics had no significant effect on the metabolic activity of bacteria belonging to the phylum Firmicutes.

Epithelial cells and immune cells are missing in *in vitro* models^15^. Addition of enterocytes and/or immune cells to an *in vitro* model will broaden the current understanding of the effect of different microbial metabolite patterns on host cells. In summary, the KUHIMM was able to capture an increase in SCFA production due to increased metabolic activity of human colonic microbiota, with no accompanying alteration in microbial composition, in response to a low-dosage supply of prebiotics. These results suggest that the positive effect of prebiotics seen *in vitro* using our human colonic microbiota model can be achieved also *in vivo* in human subjects upon administration of 6 g of prebiotics per day, although increased SCFA production is difficult to detect in the human intestine. This amount represents a realistic intake and does not pose a burden on a person’s eating habits.

## Materials and Methods

### Faecal sample collection from human volunteers

Faecal samples were obtained from eight Japanese healthy human volunteers, who had not been treated with antibiotics for more than two months prior to the experiment. All participants were recruited according to the inclusion criteria, which comprised an age of 20 to 65 years, being Japanese, non-smoking status, good health and physical condition. The exclusion criteria included significant clinical deviation from normal as determined by investigators; history or suspicion of diabetes, liver disease, kidney disease, or having a food allergy; or taking supplemental dietary fibre or lipid-lowering medications. Volunteers were designated as F26 (female, age 26), F40 (female, age 40), F63 (female, age 63), M27 (male, age 27), M37 (male, age 37), M38 (male, age 38), M39 (male, age 39), and M60 (male, age 60). All subjects provided written informed consent prior to specimen collection. The study was performed in accordance with the guidelines of Kobe University Hospital, and approved by the institutional ethics review board of Kobe University. All methods in this study were in accordance with the Declaration of Helsinki. The authors have no financial or personal relationships that could inappropriately influence this research.

Faecal samples were immediately collected in an anaerobic culture swab (212550 BD BBL Culture Swab; Becton, Dickinson and Company, Franklin Lakes, NJ, USA) and used within 24 h.

### Operation of the KUHIMM with and without prebiotics

The KUHIMM was operated using a multi-channel fermenter (Bio Jr.8; ABLE, Tokyo, Japan), as described previously^13,21^ with some modifications. The KUHIMM consisted of eight parallel and independent vessels. Each vessel contained 100 mL of Gifu Anaerobic Medium (GAM; Nissui Pharmaceutical Co., Ltd., Tokyo, Japan). The medium was autoclaved at 115 °C for 15 min and the initial pH was adjusted to 6.5. Anaerobic conditions in the vessel were achieved by purging with a mixture of N_2_ and CO_2_ (80:20; 15 mL/min) that was filter-sterilised through a 0.2-µm PTFE membrane (Pall Corporation, Port Washington, NY, USA) at 37 °C for 1 h prior to cultivation. To prepare the inoculum, the faecal sample in the swab was suspended in 2.0 mL of 0.1 M phosphate buffer (pH 6.5, consisting of 0.2 M NaH_2_PO_4_ and 0.1 M Na_2_HPO_4_) supplemented with 1.0% L-ascorbic acid (Wako Pure Chemical Industries, Osaka, Japan).

Cultivations were initiated by inoculating one faecal suspension (100 µL) into each vessel. During fermentation at 37 °C, the culture broth was stirred at 300 rpm with a magnetic stirrer and continuously purged with a filter-sterilised mixture of gas to maintain anaerobic conditions. Aliquots (1 mL) of culture broth were sampled from the vessel at 30 h after the initiation of cultivation. Faeces and culture broth samples were stored at −20 °C until use.

To evaluate the effect of prebiotics (Table 1), one type of prebiotics was added into one of the vessels at a final concentration of 2.0 g/L (0.2% per 100-mL vessel) prior to fermentation. The following prebiotics were used: DEX (Fibersol-2; Matsutani Chemical Industry Co., Ltd., Hyogo, Japan), αCD (Cyclochem Co., Ltd., Hyogo, Japan), and DXR (Dextran 40,000; Wako Pure Chemical Industries). A control vessel without prebiotics was prepared.

### Human intervention study

All participants were recruited according to the inclusion criteria, which comprised an age of 20 to 50 years, being Japanese, non-smoking status, good health and physical condition, and a fasting triglyceride level of 120 mg/dL to 199 mg/dL. The exclusion criteria included significant clinical deviation from normal as determined by investigators; history or suspicion of diabetes, liver disease, kidney disease, or having a food allergy; or taking supplemental dietary fibre or lipid-lowering medications. A human intervention study was conducted on four volunteers (F26, M37, M38, and M39) (Supplementary Fig. S2). At first, the volunteer drank water (200 mL) twice a day for 14 days (days 1–14, washout-1), and faeces were sampled using the swab on day 14. Then, the volunteer ate 3.0 g of αCD dissolved in water (200 mL) twice a day (6.0 g/day) for seven days (days 15–21), and faeces were sampled on day 21. Next, the volunteer drank water (200 mL) twice a day for 14 days (days 22–35, washout-2) again, and faeces were sampled on day 35. Thereafter, the volunteer ate 3.0 g of DEX dissolved in water (200 mL) twice a day (6.0 g/day) for seven days (days 36–42), and faeces were sampled on day 42. During the study, the four volunteers avoided drinking alcohol or consuming other dietary fibres.

The study was performed in accordance with the guidelines of Kobe University Hospital, and approved by the institutional ethics review board of Kobe University. All methods in this study were in accordance with the Declaration of Helsinki and the approved guidelines by the Medical Ethics Committee at Kobe University (research code; 290001, approved date; 21 Mar 2017). The authors have no financial or personal relationships that could inappropriately influence this research.

### Extraction of microbial genomic DNA

Microbial genomic DNA was extracted from suspended faeces and culture broth from the KUHIMM at 30 h, as described previously^21^. Purified DNA was eluted into TE buffer (10 mM TrisHCl, 1.0 mM EDTA) and stored at −20 °C until use.

### Illumina library generation

Bacterial 16S rRNA genes (V3-V4 region) were amplified using genomic DNA as template and primers S-D-Bact-0341-b-S-17 (5′-CCTACGGGNGGCWGCAG-3′) and S-D-Bact-0785-a-A-21 (5′-GACTACHVGGGTATCTAATCC-3′)^38^, as described previously^13^. Index primers (Nextera XT Index Kit; Illumina Inc., San Diego, CA, USA) overhanging the amplified sequences were added to the gene-specific sequences. The PCR reaction was performed according to the manufacturer’s instructions. Amplicons were purified with AMPure XP DNA purification beads (Beckman Coulter, Brea, CA, USA) and eluted in 25 µL of 10 mM Tris (pH 8.5). Purified amplicons were quantified using an Agilent Bioanalyzer 2100 with DNA 1000 chips (Agilent Technology, Santa Clara, CA, USA) and Qubit 2.0 (Thermo Fisher Inc., Waltham, MA, USA), and pooled in equimolar concentration (5 nM). The 16S rRNA genes along with an internal control (PhiX control v3; Illumina) were subjected to paired-end sequencing using a Miseq sequencer (Illumina) with a Miseq reagent kit v3 (600 cycles; Illumina). The PhiX sequences were removed, and paired-end reads with Q scores ≥ 20 were joined using the software package MacQIIME version 1.9.1^39^. The UCLUST algorithm^40^ was used to cluster filtered sequences into OTUs based on a ≥97% similarity threshold. Chimeric sequences were checked and removed from the library using ChimeraSlayer^41^. Representative sequences from each OTU were taxonomically classified via the GreenGenes taxonomic database using the Ribosomal Database Project Classifier^42^. OTUs were used for α-diversity estimation of the Chao1, Shannon diversity, and Simpson indexes. PCoA plots were calculated using OTUs from each sample based on unweighted UniFrac distances.

### Real-time PCR

Real-time PCR to quantify total bacterial growth during cultivation was performed using a TP700 Thermal Cycler Dice Real Time System Lite (Takara Bio Inc., Ohtsu, Japan) with a primer set targeting all eubacteria^21^. The PCR reaction and amplification were performed as described previously^21^.

### Measurement of SCFAs

Concentrations of SCFAs such as acetate, propionate, butyrate, lactate, and succinate were measured using a high-performance liquid chromatograph (HPLC; Shimadzu Corporation, Kyoto, Japan) equipped with an Aminex HPX-87H column (Bio-Rad Laboratories, Inc., Hercules, CA, USA) and a RID-10A refractive index detector (Shimadzu Corporation). The HPLC was operated at 65 °C using 5 mM H_2_SO_4_ as the mobile phase with a flow rate of 0.6 mL/min.

### Bioinformatics and statistical analyses

The various α-diversity values (Chao1, Shannon index, and Simpson index) were calculated using the MacQIIME software package^39^ because they best fit the data distribution. PCoA was conducted using OTU information from each sample and calculated based on unweighted UniFrac distances^43^ using MacQIIME. The nonparametric Kruskal-Wallis test, Dunnett test, and MANOVA were performed using JMP 13 software (SAS Institute Inc., Cary, NC, USA). *P* < 0.05 was considered statistically significant.

### Data availability

All of the raw sequence data generated in this study have been deposited on the MG-RAST server^44^ (http://metagenomics.anl.gov) as “Single Batch Fermentation System Simulating Human Colonic Microbiota_Indigestible Oligosaccharides” under accession numbers “mgm4757055.3–mgm4757122.3”.

## Electronic supplementary material

Supplementary Information
