# Supplementary material for: Low amounts of dietary fibre increase in vitro production of short-chain fatty acids without changing human colonic microbiota structure
Source: Sci Rep. 2018 Jan 11;8:435. doi: 10.1038/s41598-017-18877-8 (PMC5765155; doi:10.1038/s41598-017-18877-8)
Supplement: Supplementary file 1 — Supplementary Information [file 41598_2017_18877_MOESM1_ESM.pdf]

# Low amounts of dietary fibre increase *in vitro* production of short-chain fatty acids without changing human colonic microbiota structure

***Scientific Reports***

Daisuke Sasaki[1¶], Kengo Sasaki[1¶,*], Naoko Ikuta[2], Takahiro Yasuda[3], Itsuko Fukuda[4,5], Akihiko Kondo[1,6], Ro Osawa[4,5]

[1] Graduate School of Science, Technology and Innovation, Kobe University, 1-1 Rokkodai-cho, Nada-ku, Kobe, Hyogo 657-8501, Japan

[2] Graduate School of Medicine, Kobe University, 7-5-2 Kusunoki-cho Chuo-ku, Kobe, Hyogo 650-0017, Japan

[3] Clinical & Translational Research Center, Kobe University Hospital, 7-5-2 Kusunoki-cho Chuo-ku, Kobe, Hyogo 650-0017, Japan

[4] Department of Bioresource Science, Graduate School of Agricultural Science, Kobe University, 1-1 Rokkodai-cho, Nada-ku, Kobe, Hyogo 657-8501, Japan

[5] Research Center for Food Safety and Security, Graduate School of Agricultural Science, Kobe University, 1-1 Rokkodai-cho, Nada-ku, Kobe, Hyogo 657-8501, Japan

[6] RIKEN Center for Sustainable Resource Science, 1-7-22 Suehiro-cho, Tsurumi-ku, Yokohama, Kanagawa 230-0045, Japan

## Supplementary Table S1.

**Eubacterial copy numbers among cultures in the KUHIMM without prebiotics (CUL) and with indigestible dextrin (DEX), α-cyclodextrin (αCD), or dextran (DXR) after 30 h of fermentation.**

| | KUHIMM | | | |
|---|---|---|---|---|
| **Faecal inoculum** | **CUL** (×$10^{10}$ copies/mL) | **DEX** (×$10^{10}$ copies/mL) | **αCD** (×$10^{10}$ copies/mL) | **DXR** (×$10^{10}$ copies/mL) |
| **F26** | 6.74 | 8.75 | 9.85 | 9.85 |
| **F40** | 8.17 | 9.80 | 7.06 | 12.1 |
| **F62** | 10.2 | 12.1 | 10.7 | 12.7 |
| **M27** | 6.23 | 6.46 | 9.37 | 7.62 |
| **M37** | 7.92 | 11.0 | 6.77 | 11.0 |
| **M38** | 6.75 | 10.4 | 8.28 | 8.58 |
| **M39** | 4.69 | 7.26 | 5.74 | 6.94 |
| **M60** | 10.8 | 13.3 | 11.3 | 14.3 |

## Supplementary Figure S1.

### Transitions of total SCFA, acetate, propionate, butyrate, and ammonia concentrations during fermentation in the KUHIMM without prebiotics.

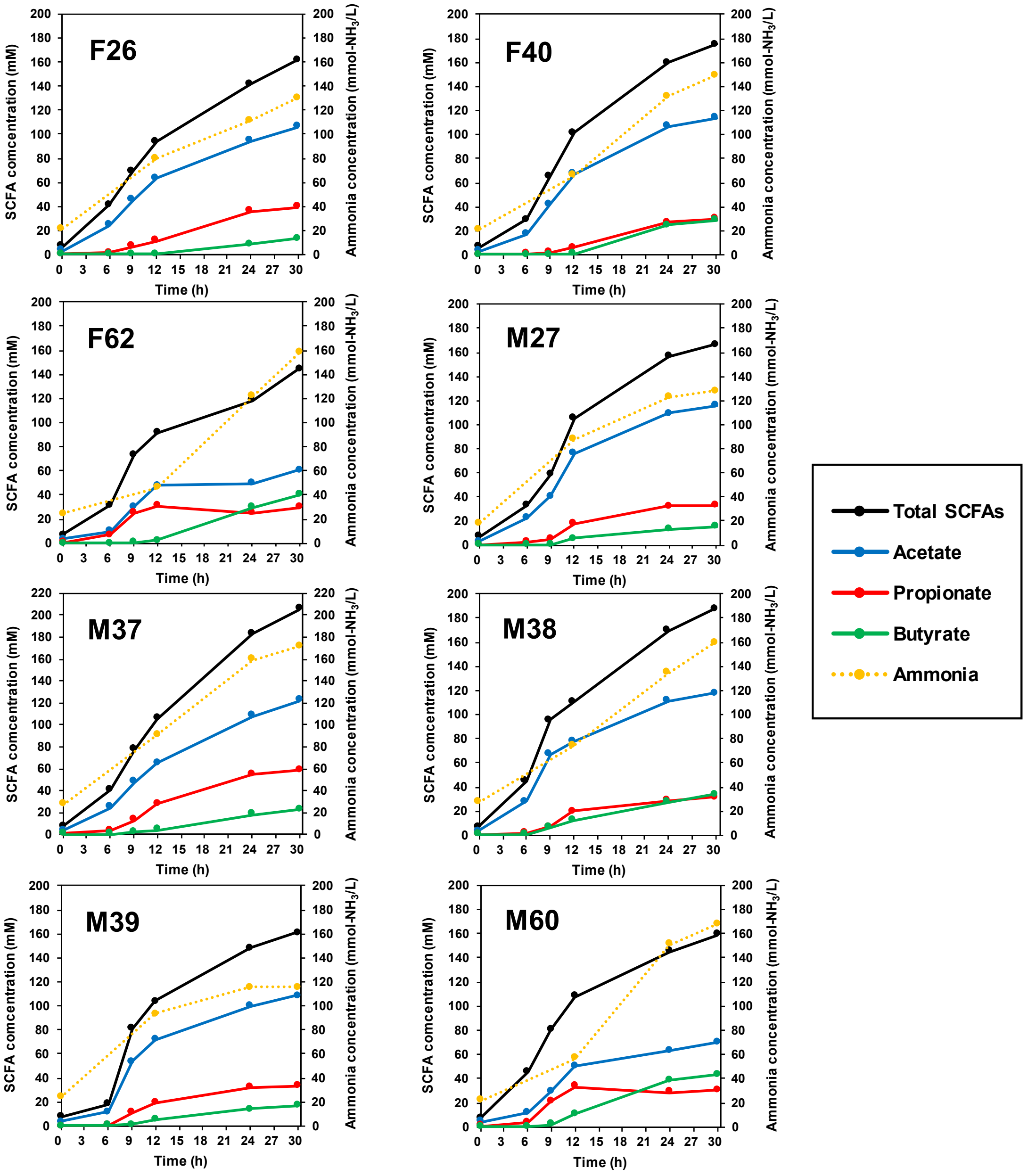


Concentrations of total SCFAs (black lines), acetate (blue lines), propionate (red lines), butyrate (green lines), and ammonia (yellow dotted lines) in the KUHIMM without prebiotics (CUL) in each human faecal sample (designated as F26, F40, F62, M27, M37, M38, M39, and M60) are shown.

## Supplementary Figure S2.

**(a) Time schedule and (b) genus-level compositional view of bacteria in human faecal samples from a human intervention study.**

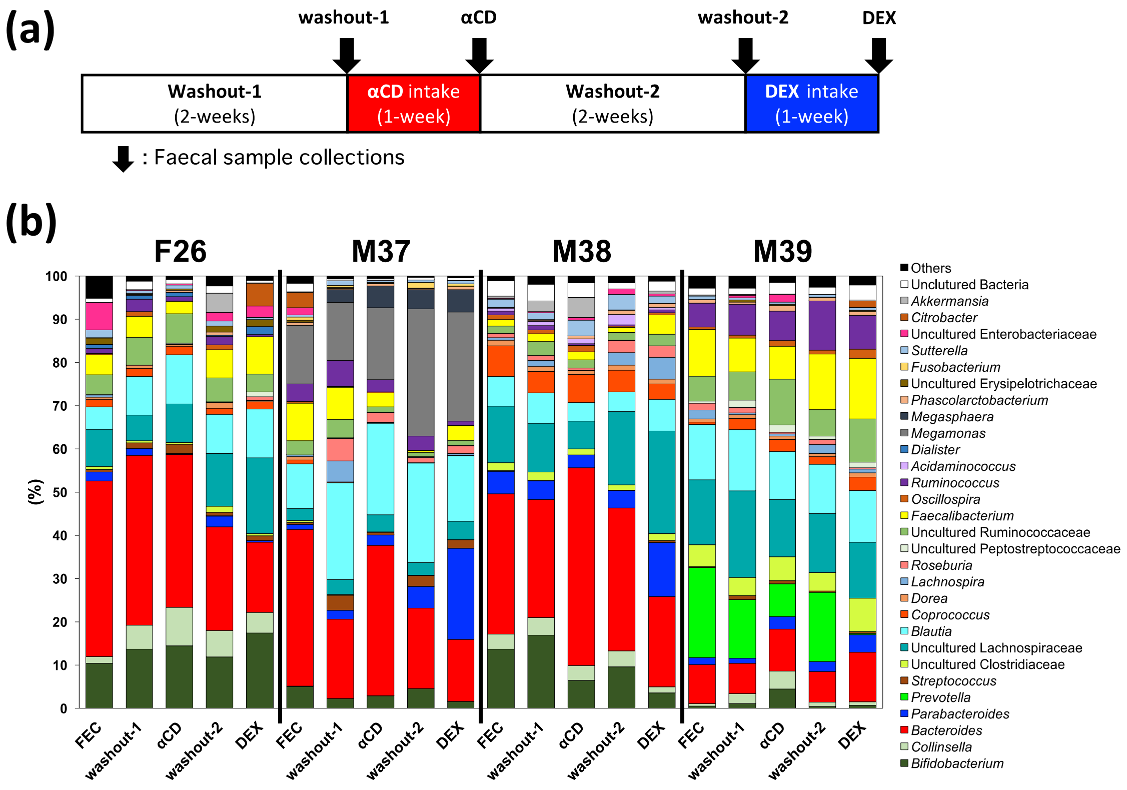


Four human volunteers (designated as F26, M37, M38, and M39) participated in this study. Each subject drank water for 14 days to calibrate the colonic microbiota composition, after which faecal samples were collected (designated as washout-1 and washout-2). Following the washout term, each subject ate α-cyclodextrin or indigestible dextrin for seven days and faecal samples were collected (designated as αCD or DEX, respectively). Bacterial genera of lower abundance (<1.0%) and lower similarity (<97%) were included in Others and Uncultured Bacteria, respectively.

## Supplementary Figure S3.

**Relationship between relative abundance (%) of each phylum and proportion (%) of each short-chain fatty acid (SCFA) relative to total SCFAs in the *in vitro* batch fermentation system, KUHIMM, after 30 h of fermentation.**

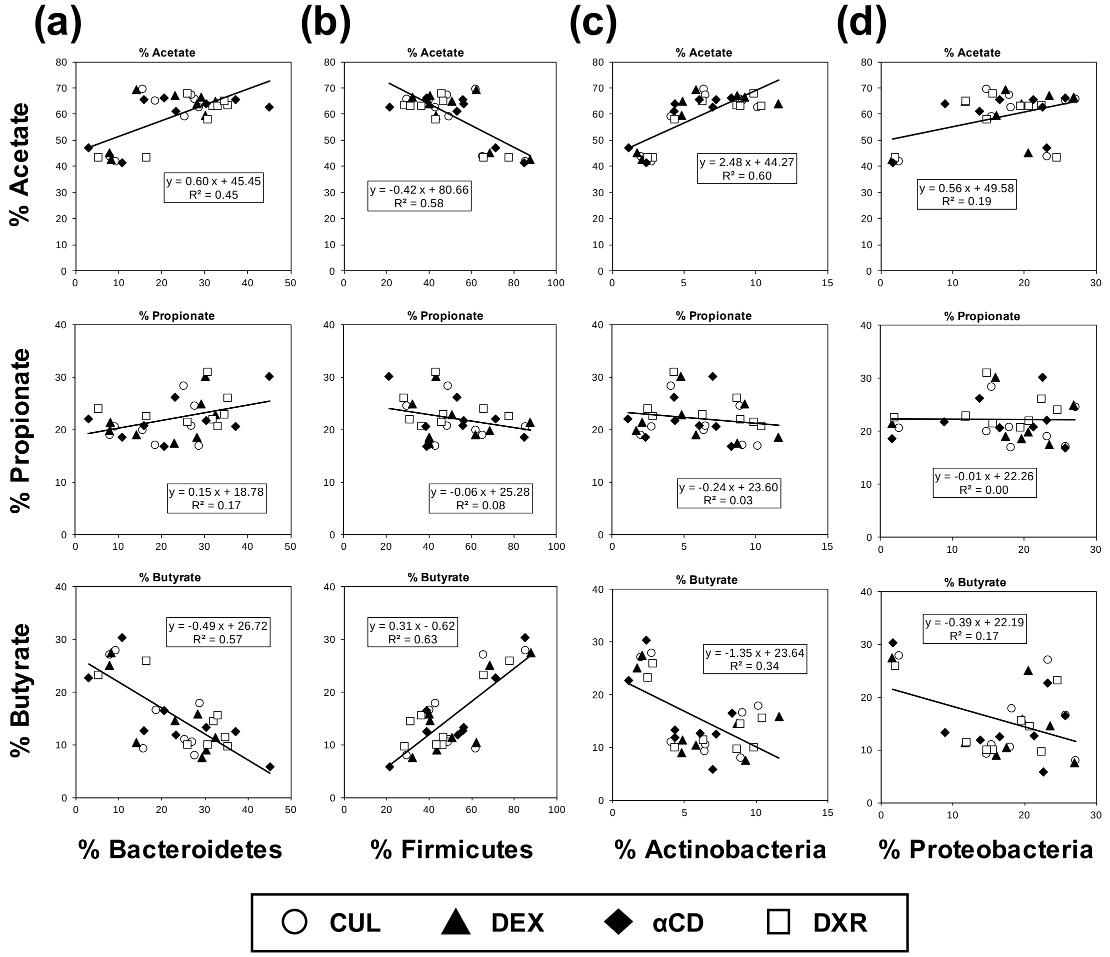


Correlations were studied for the phyla (a) Bacteroidetes, (b) Firmicutes, (c) Actinobacteria, and (d) Proteobacteria. The KUHIMM was operated without prebiotics (CUL; open circles) and with indigestible dextrin (DEX; closed triangles), α-cyclodextrin (αCD; closed diamonds), or dextran (DXR; open squares). Solid lines and the corresponding equations indicate a best-fit linear relationship.
